# Supplementary material for: Association between cigarette smoking and ovarian reserve among women seeking fertility care
Source: PLoS One. 2022 Dec 13;17(12):e0278998. doi: 10.1371/journal.pone.0278998 (PMC9746951; doi:10.1371/journal.pone.0278998)
Supplement: S1 File — (DOCX) [file pone.0278998.s001.docx]

# **Supplemental Tables**

**Table S1. Characteristics of Women in the LOUSSI Study, Stratified by Self-reported Smoking Status (N =207)**

|  | **Never smokers**  **N (%)**  **(n=150)** | **Current Smokers**  **N (%)**  **(n=33)** | **Former Smokers**  **N (%)**  **(n=24)** | **P-value** |
| --- | --- | --- | --- | --- |
| **Age (years)** |  |  |  | 0.11 |
| *21-25* | 20 (13.3) | 9 (27.4) | 4 (16.7) |  |
| *26-30* | 33 (22.0) | 8 (24.2) | 1 (4.2) |  |
| *31-35* | 46 (30.7) | 8 (24.2) | 11 (45.8) |  |
| *36-45* | 51 (34.0) | 8 (24.2) | 8 (33.3) |  |
| **Race** |  |  |  | <0.001 |
| *White* | 75 (49.0) | 17 (51.5) | 19 (79.1) |  |
| *Black* | 45 (30.2) | 15 (45.5) | 1 (4.2) |  |
| *Other* | 31 (20.8) | 1 (3.0) | 4 (16.7) |  |
| *Missing* | 1(0.7) |  |  |  |
| **Age at menarche(years) mean (s.d)** | 12.5 (1.9) | 12.0 (1.5) | 12.5 (1.3) | 0.23 |
| *Missing* | 6 (4.3) |  |  |  |
| **Body Mass Index** |  |  |  | 0.29 |
| *17.5-24.9* | 40 (27.0) | 7 (21.2) | 3 (12.5) |  |
| *25-29.9* | 47 (31.8) | 8 (24.2) | 8 (33.3) |  |
| *30-34.9* | 27 (18.2) | 12 (36.4) | 6 (25.0) |  |
| *≥ 35* | 34 (23.0) | 6 (18.2) | 7 (29.2) |  |
| *Missing* | 2 (1.3) |  |  |  |
| **Polycystic Ovary Syndrome** |  |  |  | 0.71 |
| *No* | 93 (62.0) | 18 (54.6) | 14 (58.3) |  |
| *Yes* | 57 (38.0) | 15 (45.4) | 10 (41.7) |  |
| *Missing* |  |  |  |  |
| **Parity** |  |  |  | 0.54 |
| *None* | 62 (42.2) | 10 (30.3) | 11 (45.8) |  |
| *One* | 35 (23.8) | 8 (24.2) | 7 (29.2) |  |
| *Two or more* | 50 (34.0) | 15 (45.5) | 6 (25.0) |  |
| *Missing* | 3 (2.0) |  |  |  |
| **Marital Status** |  |  |  | <0.001 |
| *Single* | 45 (31.5) | 21 (70.0) | 4 (17.4) |  |
| *Married* | 98 (68.5) | 9 (30.0) | 19 (82.6) |  |
| *Missing* | 7 (4.7) | 3 (9.1) | 1 (4.2) |  |
| **Serum AMH categories (ng/mL)** |  |  |  | 0.96 |
| **<1** | 36 (24.0) | 7 (21.2) | 5 (20.8) |  |
| **1-2** | 25 (16.6) | 7 (21.2) | 5 (20.8) |  |
| **2-3** | 43 (28.7) | 11 (33.4) | 8 (33.4) |  |
| **>3** | 46 (30.7) | 8 (24.2) | 6 (25.0) |  |
| **Diminished ovarian reserve (DOR)**  **(AMH<1 ng/mL)** |  |  |  | 0.96 |
| *No* | 114 (76.0) | 26 (78.8) | 19 (79.2) |  |
| *Yes* | 36 (24.0) | 7 (21.2) | 5 (20.83) |  |
| **NAT2 Acetylator Status** |  |  |  |  |
| Rapid/ Intermediate Acetylators | 79 (60.7) | 18 (62.1) | 9 (64.3) | 0.97 |
| Slow Acetylators | 49 (39.3) | 11 (37.9) | 5 (35.7) |  |
| *Missing* | 38 (25.3) | 1 (3.0) | 10 (41.7) |  |

**Table S2. Cotinine Levels Stratified by Self-reported Smoking Status**

|  | N | Cotinine concentration in urine (n, row%)^a^ | | |
| --- | --- | --- | --- | --- |
| Self-reported smoking status |  | <0.5 ng/mL | 0.5 – 13.9 ng/mL | ≥ 14ng/mL |
| Current smoker | 31 | 0 | 0 | 31 (100) |
| Former smoker | 22 | 10 (45.5) | 7 (31.8) | 5 (22.7) |
| Never smoker | 146 | 61 (41.8) | 63 (43.2) | 22 (15.0) |
| Total | 199 | 71 | 70 | 58 |

*^a-^Eight women did not provide urine for cotinine assay*

**Table S3.** ***NAT2* Phenotype of Women in the LOUSSI Study (N =155)**

|  | **Rapid/ Intermediate Acetylators**  **N (%)**  **(n=95)** | **Slow Acetylators**  **N (%)**  **(n=60)** | **P-value** |
| --- | --- | --- | --- |
| **Race** |  |  | 0.70 |
| *White* | 51 (53.7) | 30 (50.0) |  |
| *Black* | 23 (24.2) | 18 (30.0) |  |
| *Other* | 20 (21.1) | 12 (20.0) |  |
