# Supplementary material for: Association between cigarette smoking and ovarian reserve among women seeking fertility care
Source: PLoS One. 2022 Dec 13;17(12):e0278998. doi: 10.1371/journal.pone.0278998 (PMC9746951; doi:10.1371/journal.pone.0278998)
Supplement: Attachment — Submitted filename: Response to Reviewers.docx [file pone.0278998.s003.docx]

# **Response to Reviewers**.

**Journal Editor- Journal Requirements:**

Please review your reference list to ensure that it is complete and correct. If you have cited papers that have been retracted, please include the rationale for doing so in the manuscript text, or remove these references and replace them with relevant current references. Any changes to the reference list should be mentioned in the rebuttal letter that accompanies your revised manuscript. If you need to cite a retracted article, indicate the article’s retracted status in the References list and also include a citation and full reference for the retraction notice.

**Response:** The reference list has been reviewed and confirmed to be complete and correct. None of the papers cited in the manuscript have been retracted. Addition of three new relevant publications to the reference list was acknowledged in the rebuttal letter and revised cover letter accompanying the revised manuscript.

A paragraph referring to Table 3 was added to the manuscript and the table was moved below the paragraph to comply with the PLOS ONE submission guidelines.

**Reviewer #2:** I have carefully read the new version of this article. Authors have included the suggested changes and explanations. I believe that in its present form this article is suitable for publication.

**Response:** Thank you for your comments.

Reviewer #3: Dear Authors,

I have read and mostly appreciated your article titled Association between cigarette smoking and ovarian reserve among women seeking fertility care, in which you have laid out a somewhat thorough analysis as to the linkage between heavy smoking and poor ovarian reserve.
I believe the manuscript has strengths: it is competently structured and coherently enunciates meaningful findings. The methodology is sound, as far as I was able to determine, and leads to insightful deductions and cogently delineated conclusions; relevance, thoroughness and well-crafted structure are certainly pros with the article as well.
The weakness I should point to is the scarce reference pool and outdated sources.
Among the studies laying out the AMH-ovarianreserve-smoking correlation, which is still based on rather inconclusive data, the authors have left out several recent ones that need to be drawn upon and could contribute to better contextualization and broader scope overall.

Consider, for instance:

Bhide P, Timlick E, Kulkarni A, Gudi A, Shah A, Homburg R, Acharya G. Effect of cigarette smoking on serum anti-Mullerian hormone and antral follicle count in women seeking fertility treatment: a prospective cross-sectional study. BMJ Open. 2022 Mar 31;12(3):e049646. doi: 10.1136/bmjopen-2021-049646.

Sansone M, Zaami S, Cetta L, Costanzi F, Signore F. Ovotoxicity of smoking and impact on AMH levels: a pilot study. Eur Rev Med Pharmacol Sci. 2021 Aug;25(16):5255-5260. doi: 10.26355/eurrev_202108_26545.

I think that the article is a commendable contribution to a highly relevant research field and ought to be greenlighted for publication with minor revisions.

Sincerely.

**Response:** Thank you for your comments. The reference pool has been reviewed and updated with 3 new relevant publications on the association of smoking with ovarian reserve. These recent publications do not change the overall conclusions of the manuscript.

**New References**

53.	Bhide P, Timlick E, Kulkarni A, Gudi A, Shah A, Homburg R et al. Effect of cigarette smoking on serum anti-Mullerian hormone and antral follicle count in women seeking fertility treatment: a prospective cross-sectional study. BMJ open. 2022;12(3):e049646. doi:10.1136/bmjopen-2021-049646.

54.	Sansone M, Zaami S, Cetta L, Costanzi F, Signore F. Ovotoxicity of smoking and impact on AMH levels: a pilot study. European review for medical and pharmacological sciences. 2021;25(16):5255-60. doi:10.26355/eurrev_202108_26545.

55.	Mitchell JM, Fee N, Roopnarinesingh R, Mocanu EV. Investigating the relationship between body composition, lifestyle factors, and anti-Müllerian hormone serum levels in women undergoing infertility assessment. Irish journal of medical science. 2022. doi:10.1007/s11845-022-03148-x.
